# Supplementary material for: The Role of Procalcitonin as an Antimicrobial Stewardship Tool in Patients Hospitalized with Seasonal Influenza
Source: Antibiotics (Basel). 2023 Mar 14;12(3):573. doi: 10.3390/antibiotics12030573 (PMC10044820; doi:10.3390/antibiotics12030573)
Supplement: Supplementary file 1 [file antibiotics-12-00573-s001.zip › Supplement S1 search string_art3.pdf]

Supplement S1 search string medline 6.1.23

https://ovidsp.ovid.com/ovidweb.cgi?T=JS&NEWS=N&PAGE=main&SHAREDSEARCHID=3g3bBRYoBW8iQ0grFzxTtIPmVQthC3z3EOeBngdS4Bn4g62fWMUDM0iSga6e8JR0y

1

Procalcitonin/

1666

Advanced

Display Results

More

2

("PCT" or "Procalcitonin" or "calcitonin*" or "pro-calcitonin").tw.

40110

Advanced

Display Results

More

3

1 or 2

40200

Advanced

Display Results

More

4

Diagnosis/ or Clinical Decision-Making/ or Diagnosis, Differential/ or Early Diagnosis/ or Prognosis/

1084487

Advanced

Display Results

More

5

("Diagnos*" or "decision*" or "decide*").tw.

3350486

Advanced

Display Results

More

6

4 or 5

4040896

Advanced

Display Results

More

7

Antimicrobial Stewardship/

3201

Advanced

Display Results

More

8

("Antibiotic Stewardship" or "Antimicrobial Stewardship" or "antibiotic*").tw.

397644

Advanced

Display Results

More

9

7 or 8

397992

Advanced

Display Results

More

10

Influenza, Human/

56618

Advanced

Display Results

More

11

("Influenz*" or "flu").tw.

141509

Advanced

Display Results

More

12

10 or 11

148144

Advanced

Display Results

More

13

inpatients/

28308

Advanced

Display Results

More

14

("inpatient*" or "hospitali*").tw.

426055

Advanced

Display Results

More

15

13 or 14

432924

Advanced

Display Results

More

16

6 or 9

4368650

Advanced

Display Results

More

17

3 and 12 and 15 and 16

31

Advanced

Display Results

More

18

3 and 12 and 16

106

Advanced

Display Results

More

19

remove duplicates from 18

106

Advanced

Display Results

More

20

limit 19 to last 10 years

78

Advanced

Display Results

More

After reading the **titles** the following articles were on influenza and procalcitonin as an inflammatory /diagnostic marker

1. ***93. Jiali D., Yuejie Y., Shuya H., et al Clinical characteristics and prognostic factors of adult patients with acute respiratory failure due to influenza infection. [in Chinese] Zhonghua Wei Zhong Bing Ji Jiu Yi Xue 2020;32(11):1304-1309. doi:10.3760/cma.j.cn121430-20200827-00598

2. ***104. Gautam S., Cohen A.J., Stahl Y., et al Severe respiratory viral infection induces procalcitonin in the absence of bacterial pneumonia. Thorax 2020;75(11):974-981. doi:10.1136/thoraxjnl-2020-214896

3. *** 105. Houying Q., Mengdie L., Ling Z., Hui Z. Clinical characteristics and prognosis analysis of 37 patients with severe influenza. Zhonghua Wei Zhong Bing Ji Jiu Yi Xue 2020;32(10):1253-1256. doi:10.3760/cma.j.cn121430-20200428-00344

4. *** 132. Estella A., Martin Cano J.M., Garrino A., et al Retrospective comparative study with historical control between 2009 pandemic influenza H1N1 viral infections, 2010-2011 influenza season and SARS COV 2 infection in an ICU of a community hospital. Intensive Care Med. Exp. 2020;8(SUPPL 2):no pagination. doi:10.1186/s40635-020-00354-8

5. *** 146. Li Z., He L., Li S., et al Combination of procalcitonin and C-reactive protein levels in the early diagnosis of bacterial co-infections in children with H1N1 influenza. Influ. Other Respir. Viruses 2019;13(2):184-190. doi:10.1111/irv.12621

6. *** 154. O'Riordan F., Shiely F., Byrne S., et al An investigation of the effects of procalcitonin testing on antimicrobial prescribing in respiratory tract infections in an Irish university hospital setting: A feasibility study. J. Antimicrob. Chemother. 2019;74(11):3352-3361. doi:10.1093/jac/dkz313

7. *** 165. Lee C.C., Chia-Yu C., Xiao-Wei M., et al Combining procalcitonin and rapid multiplex respiratory virus testing for antibiotic stewardship in elderly patients with severe acute respiratory infection. Intensive Care Med. Exp. 2019;7(Supplement 3):no pagination. doi:10.1186/s40635-019-0265-y

8. *** 170. Politis P.A., Kallstrom G., Tan M., File T.M. Early discontinuation of antibacterials is safe for patients with community-acquired pneumonia (CAP) who have a positive viral test, negative tests for bacteria, and low procalcitonin. Open Forum Infect. Dis. 2019;6(Supplement 2):S765. doi:10.1093/ofid/ofz360.1915

9. *** 197. Taymaz T., Ergonul O., Kebapci A., Okyay R. Significance of the detection of influenza and other respiratory viruses for antibiotic stewardship: Lessons from the post-pandemic period. Int. J. Infect. Dis. 2018;77:53-56. doi:10.1016/j.ijid.2018.10.003

10. *** 199. Canavaggio P., Boutolleau D., Goulet H., Riou B., Hausfater P. Procalcitonin for clinical decisions on influenza-like illness in emergency department during influenza a(H1N1)2009 pandemic. Biomarkers 2018;23(1):10-13. doi:10.1080/1354750X.2016.1276626

11. *** 239. Yang M., Gao H., Chen J., et al Bacterial coinfection is associated with severity of avian influenza A (H7N9), and procalcitonin is a useful marker for early diagnosis. Diagn. Microbiol. Infect. Dis. 2016;84(2):165-169. doi:10.1016/j.diagmicrobio.2015.10.018

12. *** 243. Henriquez K.M., Hayney M.S., Rakel D.P., Barrett B. Procalcitonin levels in acute respiratory infection. Viral Immunol. 2016;29(2):128-131. doi:10.1089/vim.2015.0106

13. *** 246. McDonagh M.S., Peterson K., Winthrop K., Cantor A., Holzhammer B., Buckley D. Systematic evaluation of interventions to reduce overprescribing of antibiotics for acute respiratory tract infections. Pharmacoepidemiol. Drug Saf. 2016;25(Supplement 3):387-388. doi:10.1002/pds.4070

14. *** 260. Fitch G., Etherington C., Whitaker P., Bosomworth M., Peckham D. Procalcitonin and C reactive protein levels in hospitalised patients receiving intravenous antibiotics. J. Cyst. Fibrosis 2015;14(SUPPL. 1):S65. Cited in: Embase at http://ovidsp.ovid.com/ovidweb.cgi?T=JS&PAGE=reference&D=emed16&NEWS=N&AN=71951684. Accessed August 30, 2022.

15. *** 268. Pfister R., Kochanek M., Leygeber T., et al Procalcitonin for diagnosis of bacterial pneumonia in critically ill patients during 2009 H1N1 influenza pandemic: A prospective cohort study, systematic review and individual patient data meta-analysis. Crit. Care 2014;18(2):no pagination. doi:10.1186/cc13760

16. *** 279. Joseph C., Togawa Y., Shindo N. Bacterial and viral infections associated with influenza. Influ. Other Respir. Viruses 2013;7(SUPPL.2):105-113. doi:10.1111/irv.12089

17. *** 284. Wu M.-H., Lin C.-C., Huang S.-L., et al Can procalcitonin tests aid in identifying bacterial infections associated with influenza pneumonia? A systematic review and meta-analysis. Influ. Other Respir. Viruses 2013;7(3):349-355. doi:10.1111/j.1750-2659.2012.00386.x

18. *** 300. Paiva M.B.S., Botoni F.A., Teixeira Jr. A.L., et al The behavior and diagnostic utility of procalcitonin and five other inflammatory molecules in critically ill patients with respiratory distress and suspected 2009 influenza a H1N1 infection. Clinics 2012;67(4):327-334. doi:10.6061/clinics/2012%2804%2905

19. *** 318. Teng F, Wan TT, Guo SB, et al. Outcome prediction using the Mortality in Emergency Department Sepsis score combined with procalcitonin for influenza patients. Med Clin (Barc). 2019;153(11):411-417. doi:10.1016/j.medcli.2019.03.021, 10.1016/j.medcli.2019.03.021

20. *** 322. Suarez NM, Bunsow E, Falsey AR, Walsh EE, Mejias A, Ramilo O. Superiority of transcriptional profiling over procalcitonin for distinguishing bacterial from viral lower respiratory tract infections in hospitalized adults. J Infect Dis. 2015;212(2):213-22. doi:10.1093/infdis/jiv047, 10.1093/infdis/jiv047

21. Procalcitonin in patients with influenza A (H1N1) infection and acute respiratory failure.

22. Duarte PA, Bredt CS, Bredt GL Jr, Jorge AC, Venazzi A, Tondo LG, Oliveira LS, Jorge MM, Marchiori R, Giancursi TS, Coradin M, Alexandrino AG.

23. Procalcitonin and C-reactive protein in severe 2009 H1N1 influenza infection.

Ingram PR, Inglis T, Moxon D, Speers D.

24. A Comparative Systematic Review of COVID-19 and Influenza.

Osman M, Klopfenstein T, Belfeki N, Gendrin V, Zayet S. – COMPARE FLU AND COVID-19

25. Procalcitonin in children with suspected novel influenza A (H1N1) infection. Limper M, Smit PM, Bongers KM, van Zanten AP, Smits PH, Brandjes DP, Mulder JW, von Rosenstiel IA, van Gorp EC.

26. The utility of procalcitonin in diagnosis of H1N1 influenza in intensive care patients. Hammond NE, Corley A, Fraser JF.

27. Low Levels of Procalcitonin Are Related to Decreased Antibiotic Use in Children Hospitalized Due to Influenza. Wrotek A, Wrotek O, Jackowska T.

28. Significance of high levels of procalcitonin in patients with influenza A (H1N1) pneumonia. Guervilly C, Coisel Y, Botelho-Nevers E, Dizier S, Castanier M, Lepaul-Ercole R, Brissy O, Roch A, Forel JM, Papazian L.

29. Optimal use of procalcitonin to rule out bacteremia in patients with possible viral infections. Azijli K, Minderhoud TC, de Gans CJ, Lieveld AWE, Nanayakkara PWB.J Am Coll Emerg Physicians Open. 2022 May 19;3(3):e12621. doi: 10.1002/emp2.12621. eCollection 2022 Jun.

30. Using procalcitonin-guided algorithms to improve antimicrobial therapy in ICU patients with respiratory infections and sepsis. Schuetz P, Raad I, Amin DN. Curr Opin Crit Care. 2013 Oct;19(5):453-60. doi: 10.1097/MCC.0b013e328363bd38.

31. Procalcitonin levels are lower in intensive care unit patients with H1N1 influenza A virus pneumonia than in those with community-acquired bacterial pneumonia. A pilot study.Piacentini E, Sánchez B, Arauzo V, Calbo E, Cuchi E, Nava JMJ Crit Care. 2011 Apr;26(2):201-5. doi: 10.1016/j.jcrc.2010.07.009. Epub 2010 Sep 1. PMID: 20813489

32. Can procalcitonin help identify associated bacterial infection in patients with severe influenza pneumonia? A multicentre study. Cuquemelle E, Soulis F, Villers D, Roche-Campo F, Ara

Somohano C, Fartoukh M, Kouatchet A, Mourvillier B, Dellamonica J, Picard W, Schmidt M, Boulain T, Brun-Buisson C; A/H1N1 REVA-SRLF Study Group.

33. Role of procalcitonin and C-reactive protein in differentiation of mixed bacterial infection from 2009 H1N1 viral pneumonia.
Ahn S, Kim WY, Kim SH, Hong S, Lim CM, Koh Y, Lim KS, Kim W.
Influenza Other Respir Viruses. 2011 Nov;5(6):398-403. doi: 10.1111/j.1750-2659.2011.00244.x. Epub 2011 Mar 30.

34. Procalcitonin (PCT) levels for ruling-out bacterial coinfection in ICU patients with influenza: A CHAID decision-tree analysis.
Rodríguez AH, Avilés-Jurado FX, Díaz E, Schuetz P, Trefler SI, Solé-Violán J, Cordero L, Vidaur L, Estella Á, Pozo Laderas JC, Socias L, Vergara JC, Zaragoza R, Bonastre J, Guerrero JE, Suberviola B, Cilloniz C, Restrepo MI, Martín-Loeches I; SEMICYUC/GETGAG Working Group.
J Infect. 2016 Feb;72(2):143-51. doi: 10.1016/j.jinf.2015.11.007. Epub 2015 Dec 15.
