# Supplementary material for: The Role of Procalcitonin as an Antimicrobial Stewardship Tool in Patients Hospitalized with Seasonal Influenza
Source: Antibiotics (Basel). 2023 Mar 14;12(3):573. doi: 10.3390/antibiotics12030573 (PMC10044820; doi:10.3390/antibiotics12030573)
Supplement: Supplementary file 1 [file antibiotics-12-00573-s001.zip › supplement S2_tentative adm.dg.pdf]

Supplement S2

**Table S1: Tentative admission diagnoses**	Number of patients
Seasonal influenza	18
Bacterial infection (chronic obstructive pulmonary disease exacerbation, pneumonia, sepsis, uro-sepsis, infection of unknown origin)	34
Airway symptoms, not specified	35
Other: none of the above (airway symptoms combined with other diagnosis: impaired general condition, pulmonary edema, symptoms from gastrointestinal, CNS, cardiovascular- or urinary system, cancer, and causa socialis)	29
Total:	116
