# Supplementary material for: The Role of Procalcitonin as an Antimicrobial Stewardship Tool in Patients Hospitalized with Seasonal Influenza
Source: Antibiotics (Basel). 2023 Mar 14;12(3):573. doi: 10.3390/antibiotics12030573 (PMC10044820; doi:10.3390/antibiotics12030573)
Supplement: Supplementary file 1 [file antibiotics-12-00573-s001.zip › Supplement S3_statistics.pdf]

Statistics used in the article “The Role of Procalcitonin as an Antimicrobial Stewardship Tool in Patients Hospitalized with Seasonal Influenza”

Codebook:

Variable	Label
ID	nnid
inndia_kateg	Admission tentative diagnosis
sympt_dager	Days of symptoms before influenza test
rtgpos	Chest X ray infiltrate
crb652	CRB_65 score
leu1/crp1/pct1	value day 1 (pluss/minus 2 dager)
leu3/crp3/pct3	value day 3 (pluss/minus 2 dager)
leu5/crp5/pct5	value day 5 (pluss/minus 2 dager)
lut_put	lut/put positiv?
utdod	Dead by hospital release
tidinnlagt	Days of hospital stay
dodsarsak	Cause of death
avd	Ward (longest stay)
prehospAB	antibiotika FØR innleggelse?
pct1_0,25diko	pct1 over /under 0,25
ddd	defined daily doses of antibiotika
haddd	hospital adjusted DDD
dot	days of therapy
ab_dag1/3/5	antibiotika på dag 1/3/5 fra inklusjonsdato (pluss/minus 2 dager)
mors3mnd	Dead by 3 måneder?
mors14d	Dead by 14 dager?
intensiv	intensiv eller overvåkning i løpet av opphold?
crp1_100diko	CRP dag 1 over 100?
crp1_50diko	CRP dag 1 over 50?
leu1_diko	leukocytter dag 1 utenfor eller innenfor referanseverdi (3,5-11)?
inkltilPCT1	dager fra innleggelse til PCT1 tatt (ekskludert de med mer enn to dager)
esk_deeskInflu	eskalert eller deeskalert etter inkludert i studiet; dvs positiv influensaprøve
esk_deeskPCT1	eskalert eller deeskalert etter første PCT svar forelå
ab1/3/5smalbred	AB dag 1/3/5 smal eller bred
ab	antibiotika dikotom
kjonn	Female/male
lutput	positiv?
baktinfmikro	Bacterial coinfection (positiv bloodkulture and/or LUT/PUT
crb652_diko	crb 65 score

Stata syntax over regression models

*table 2 , logistic regression

*test multicollinearity

```
logistic intensiv i.Kjønn Alder pct1 symptomdager
regress intensiv i.Kjønn Alder pct1 symptomdager
vif
regress intensiv i.Kjønn Alder pct1 symptomdager AB
vif
regress intensiv i.Kjønn Alder pct1 symptomdager AB DOT
vif
regress intensiv i.Kjønn Alder pct1 symptomdager AB DOT DDD
vif
regress intensiv i.Kjønn Alder pct1 symptomdager AB DOT DDD crp1
vif
regress intensiv i.Kjønn Alder pct1 symptomdager AB DOT DDD crp1 leu1
vif
regress baktinf_mikro Kjønn Alder crp1 pct1 leu1
vif
regress intensiv pct1 DDD haDDD
vif
```

*** log regression:**

* “forced” model, clinical judgement and directed acyclic graph (DAG)

* intensiv as dependent variable:

*ddd is a collider (both dependent (intensiv) and independent/explanatory (pct) variables points to ddd in a directed acyclic graph.

* therefore we omit ddd.

*I adjust for crb65, baktinf_mikro and rtgpos because these are confounders on DAG (arrow to both pct and intensiv)

* crb65 not included as collinearity with alder and had lower significance than alder in the first syntax (logistic intensiv alder pct1 baktinf_mikro kjonn crb65_2 rtgpos)

Final syntax:

logistic intensiv alder pct1 baktinf_mikro kjonn rtgpos

* mors30days as dependent v

*crp1 was a collider (i.e. preterminals have increased crp and when PCT is increased crp would co-correlate (but not necessarily vice versa)

* both death and pct points to crp in DAG

* baktinf, crb65 and rtg pos deemed as confounders, thus we adjust for these.

logistic mors3mnd alder kjonn pct1 rtgpos crb65_2 baktinf_mikro

* as age and CRB65 are dependent we remove crb65_2 as it had the lowest significance (vs alder) and final syntax is:

logistic mors3mnd alder kjonn pct1 rtgpos baktinf_mikro

*** linear regression**

*assumption 1 (linear relationship) and 5 (normally distributed):

hist ddd

bro

```
gen logddd=log(ddd)

hist logddd

hist pct1

gen logpct1=log(pct1)

hist logpct1

scatter logddd logpct1

scatter ddd pct1

hist dot

gen logdot= log(dot)

hist logdot

hist tidinnlagt

gen logtidinnlagt = log(tidinnlagt)

hist logtidinnlagt

*tidinnlagt not a confounder (thus not adjusted for)

scatter logddd logtidinnlagt

scatter logtidinnlagt logpct1

hist crp1

gen logcrp1=log(crp1)

hist logcrp1

scatter logcrp1 logpct1

*crp1 not a confounder

hist leu1

qnorm leu1

hist sympt_dager

gen logsympt_dager= log(sympt_dager)

hist logsympt_dager

scatter logsympt_dager log pct1

scatter logsympt_dager logpct1


* categorical variables relevant to adjust for (confounders): baktinf_mikro, rtgpos, crb65
```

* no multicollinearity

regres ddd pct1 tidinnlagt crp1 leu1

vif

* autocorrelation (not relevant - no time series)

bro

gen trend = _n

bro

tsset trend

reg logddd logpct1 logcrp1 leu1

reg logddd logpct1 logcrp1 leu1

predict uhat, residual

tsline uhat

tsline uhat if e(sample)==1, yline(0)

reg logddd logpct1 logcrp1 leu1 logtidinnlagt

estat dwatson

*** linear regression**

Defined daily therapy as endpoint/dependent variable:

reg logddd logpct1 rtgpos crb65_2 baktinf_mikro alder kjonn

* rtg, baktinf_mikro, CRB65_2 are confounders, and we have adjusted for them, and adjusted for age and sex.

* due to dependence of alder and CRB-65, we do not include age in the model, due to clinical judgement, as well as lowest significance (of age and CRB65) -final syntax:

reg logddd logpct1 rtgpos crb65_2 baktinf_mikro kjonn

Days of therapy as endpoint/dependent variable:

reg logdot logpct1 rtgpos crb65_2 baktinf_mikro alder kjonn

* due to dependence of alder and CRB-65, we do not include age in the model, due to clinical judgement, as well as lowest significance (of age and CRB65) -final syntax:

reg logdot logpct1 rtgpos crb65_2 baktinf_mikro kjonn
